# School-Based Physical Activity, Cognitive Performance and Circadian Rhythms: Rethinking the Timing of Movement in Education

**DOI:** 10.3390/children12101324

**Published:** 2025-10-02

**Authors:** Francesca Latino, Francesco Tafuri, Mariam Maisuradze, Maria Giovanna Tafuri

**Affiliations:** 1 Department of Education and Sport Sciences, Pegaso University, 80143 Naples, Italy; 2 Department of Literature and Cultural Heritage, University of Campania “L. Vanvitelli”, 81100 Caserta, Italy; 3 Department of Medical, Motor and Wellness Sciences, University of Naples “Parthenope”, 80133 Napoli, Italy; 4 Department of Literary, Linguistic and Philosophical Studies, Pegaso University, 80143 Naples, Italy

**Keywords:** executive functions, academic outcomes, physical education, educational neuroscience

## Abstract

**Background.** Physical activity enhances cognitive performance in adolescents, yet the role of circadian timing within the school day remains poorly understood. **Purpose.** This study examined whether the timing of school-based physical activity (morning, midday, afternoon) influences cognitive performance, subjective alertness, and mood states in early adolescents. **Methods.** A 12-week crossover intervention was conducted with 102 students (aged 12–13 years) from southern Italy. Each class participated in three 4-week conditions of structured physical activity scheduled in the morning (8:10–9:10), midday (12:10–13:10), and afternoon (15:10–16:10), separated by one-week washouts. Cognitive outcomes (d2-R, Digit Span backward, TMT-A), subjective alertness (KSS), and mood (PANAS-C) were assessed at baseline and after each condition. Analyses employed linear mixed-effects models and repeated-measures ANOVAs, adjusting for sex, BMI, chronotype, and sleep duration. **Results.** Morning activity produced the strongest improvements in attention (d2-R, η^2^p = 0.16), working memory (Digit Span backward, η^2^p = 0.06), processing speed (TMT-A, η^2^p = 0.08), alertness (KSS, η^2^p = 0.19), and positive affect (PANAS-C, η^2^p = 0.05). Midday sessions yielded moderate benefits (d2-R, η^2^p = 0.09; Digit Span backward, η^2^p = 0.05; TMT-A, η^2^p = 0.07; KSS, η^2^p = 0.09), while afternoon activity showed the weakest or nonsignificant changes (all η^2^p < 0.05). Chronotype moderated the effects on attention and working memory, with morning types deriving the largest gains. **Conclusions.** The timing of physical activity is a critical determinant of its cognitive and affective benefits. Incorporating morning exercise into school timetables may represent a low-cost, scalable strategy to optimize both learning readiness and well-being in adolescents.

## 1. Introduction

The relationship between physical activity and cognitive performance in children and adolescents has been extensively investigated over the past two decades, with growing evidence indicating that engaging in regular movement contributes not only to physical health but also to academic achievement and psychological well-being [1]. Traditional research has focused primarily on the type, frequency, and intensity of exercise, demonstrating that both acute bouts and chronic engagement in physical activity can enhance executive functions, attention, and memory, which are central to learning processes [2,3]. However, a critical and often overlooked dimension in this line of inquiry is the temporal aspect of physical activity, specifically the circadian context in which exercise occurs. Human cognitive functioning follows a biological rhythm regulated by the circadian system, which governs fluctuations in arousal, attention, and performance across the day. This suggests that the timing of physical activity might be as important as its quantity and quality, yet this factor has received little systematic exploration in the school setting [4].

Circadian rhythms are endogenous, approximately twenty-four-hour cycles that regulate physiological and behavioral processes [5]. They are primarily driven by the suprachiasmatic nucleus of the hypothalamus, which acts as a central pacemaker, synchronizing peripheral clocks distributed throughout the body. These rhythms influence numerous functions, including hormonal secretion, body temperature, sleep–wake cycles, and cognitive performance. In children and adolescents, circadian rhythms undergo developmental changes that significantly affect their learning capacity and daily functioning [6]. During puberty, for example, a well-documented delay in circadian phase occurs, leading many adolescents to experience increased eveningness and difficulty with early morning wakefulness [7]. This misalignment between biological rhythms and school schedules often results in reduced sleep duration, increased daytime sleepiness, and compromised cognitive performance in morning hours. Against this backdrop, the interaction between physical activity and circadian biology becomes particularly salient, as exercise is known to act as a powerful time cue, capable of modulating circadian rhythms and influencing alertness levels [8]. Moreover, emerging evidence suggests that the cognitive benefits of physical activity are mediated by neurobiological processes, such as increased cerebral blood flow, neurotransmitter release, and the upregulation of brain-derived neurotrophic factor, that are themselves sensitive to circadian variations in hormones and neural activity [9,10]. This implies that the timing of exercise may critically shape its cognitive outcomes, not only in relation to biological signals like cortisol and melatonin, but also with respect to individual chronotype differences, which determine whether students function more effectively in the morning or later in the day [11,12].

Despite the extensive literature on the benefits of physical activity for learning, most studies have neglected to examine when during the school day exercise might yield optimal cognitive outcomes [13]. Research on so-called “active breaks” or school-based interventions has demonstrated short-term improvements in attention and classroom behavior, yet these studies rarely consider the circadian timing of the intervention. The implicit assumption has often been that physical activity is beneficial regardless of when it occurs, but this assumption fails to acknowledge the dynamic nature of cognitive resources and biological rhythms [14]. For instance, empirical studies in chronobiology, many of which are derived from animal experiments, as well as findings in educational neuroscience, indicate that attention and working memory tend to peak in mid to late morning [15]. In contrast, post-lunch periods are consistently associated with a dip in alertness and performance in adults, whereas studies in school-aged children have yielded inconclusive results [16]. Afternoon hours, on the other hand, can produce variable outcomes depending on individual chronotype and sleep history [17]. This temporal variability raises the question of whether physical activity could mitigate circadian-related cognitive troughs or, conversely, potentiate existing peaks, thus enhancing learning outcomes in a time-dependent manner [18].

Understanding the interaction between exercise timing and cognition is particularly relevant for students in early adolescence, such as those in lower secondary school, who face increasing academic demands during a phase of profound biological and psychological transformation. At ages twelve to thirteen, students are navigating the onset of puberty, a shift in sleep patterns toward evening preference, and heightened sensitivity to environmental and lifestyle factors that influence circadian rhythms. These changes often clash with the rigid structure of school schedules, which typically begin early in the morning and may extend into the afternoon, especially in systems with full-day or extended curricula [19]. The resulting misalignment has been associated with decreased alertness, poorer academic performance, and elevated stress. By systematically examining the role of physical activity at different times of the school day, educators and researchers can identify strategies to align movement with circadian dynamics, potentially improving not only cognitive outcomes but also students’ overall educational experience [20].

From a pedagogical perspective, the exploration of circadian timing and physical activity in schools challenges the conventional organization of the school day, which is typically structured for administrative convenience rather than biological optimization [21]. If research confirms that certain times of day are more conducive to learning when preceded by physical activity, this could support evidence-based modifications to timetables, such as incorporating movement sessions in the morning to prime students for core subjects, or strategically placing them after lunch to counteract cognitive fatigue. Such changes could foster not only better academic performance but also greater student engagement and motivation. Moreover, considering timing in physical activity interventions contributes to a broader educational philosophy that values alignment between human biology and institutional practices, promoting a holistic approach to student well-being [22].

Although there is strong theoretical rationale for investigating the timing of physical activity, empirical studies remain scarce [23]. Most existing research in school settings has been conducted in the morning, often due to logistical constraints, leaving midday and afternoon sessions relatively underexplored [24]. A handful of studies outside the school context suggest that afternoon exercise may produce larger improvements in working memory and processing speed, while morning exercise may better support attention and inhibitory control [25]. However, these findings are inconsistent and limited in scope, underscoring the need for systematic, longitudinal investigations in real-world educational environments. Importantly, studies should not only measure cognitive outcomes immediately following exercise but also examine whether repeated engagement in physical activity at specific times leads to cumulative benefits over weeks or months.

The present study was therefore designed to address this gap by examining whether the timing of school-based physical activity, conducted in the morning, midday, or afternoon, differentially influences cognitive functioning, subjective alertness, and emotional states in early adolescents. Employing a crossover design with both acute and longitudinal assessments, and accounting for individual differences such as chronotype, sleep-related factors, and daily activity habits, the study aims to provide empirical evidence on how exercise timing shapes learning readiness and psychosocial well-being during this critical developmental stage.

## 2. Materials and Methods

### 2.1. Study Design

This investigation adopted an experimental crossover design with three timing conditions, conducted in lower secondary schools with students aged twelve to thirteen years. Each participating class was randomly assigned to a different sequence of intervention timings, ensuring a balanced distribution across the three conditions. The study examined the effects of school-based physical activity delivered at three distinct circadian phases: early morning (Timing A 08:10–09:10, during the first lesson), midday (Timing B 12:10–13:10, corresponding to the fifth lesson), and afternoon (Timing C 15:10–16:10, within the extended school schedule). Each condition lasted four consecutive weeks, and during each phase students engaged in two sessions per week of structured physical activity, for a total of eight sessions per condition. To reduce the likelihood of carry-over effects between conditions, a one-week washout period without the experimental intervention was scheduled between phases, during which pupils continued with their regular physical education classes.

The intervention covered a total duration of fourteen weeks, beginning in January 2025 and concluding in April 2025. All procedures were conducted in accordance with the ethical standards outlined in the Declaration of Helsinki and its subsequent amendments. Ethical approval for the study protocol was granted by the Department of Medical, Motor, and Wellness Sciences at the University of Naples “Parthenope” (DiSMMeB Prot. N. 88592/2024). Participation was voluntary, and both students and their parents or legal guardians received detailed information regarding the aims, procedures, and potential benefits and risks of the study. Written informed consent was obtained from parents or guardians, and students provided assent prior to participation.

**Figure 1:**
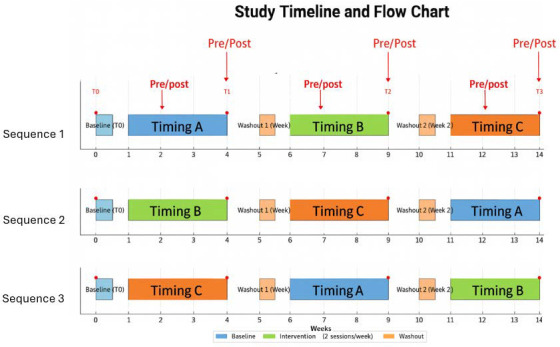
Schedule of the crossover study. The intervention lasted 14 weeks and consisted of three 4-week phases of school-based physical activity carried out at different circadian timings (Timing A: morning, Timing B: midday, Timing C: afternoon), separated by one-week washout periods. Cognitive assessments were conducted at baseline (T0) and at the end of each intervention phase (T1, T2, T3 in the Morning, 8:30–10:00 a.m.). Additional pre–post cognitive tests were administered during Weeks 2, 4, 7, 9, 12, and 14 to capture acute effects.

The crossover design (Figure 1) ensured that each participant experienced all three timing conditions, thereby controlling for inter-individual differences and strengthening the reliability of comparisons across circadian phases. Cognitive assessments were conducted acutely, immediately before and after selected activity sessions, and longitudinally, at the beginning and end of each four-week condition. In the longitudinal design, four measurement points were defined: T0 (baseline, before the start of the first condition), T1 (end of the first four-week condition), T2 (end of the second four-week condition), and T3 (end of the third four-week condition). These time points referred specifically to the administration of cognitive tests, while acute pre–post assessments were carried out during Weeks 2, 4, 7, 9, 12, and 14. This dual approach allowed the study to capture both short-term and cumulative effects, providing a comprehensive understanding of the interaction between circadian rhythms, school-based physical activity, and cognitive performance in adolescents.

### 2.2. Participants

Participants (Table 1) were recruited from two lower secondary schools in Southern Italy, both of which operated under an extended day curriculum with afternoon classes twice per week. The target population consisted of students enrolled in the second year of lower secondary education, corresponding to an age range of 12 to 13 years. Four intact classes per school were invited to participate, yielding a potential pool of approximately 120 students.

A priori sample size estimation was conducted using G*Power 3.1, assuming a repeated-measures within–between interaction design with three conditions (morning, midday, afternoon) and four measurement points (baseline and the end of each intervention period). An effect size of f = 0.20 (small-to-medium, based on prior literature on acute physical activity and cognitive performance in school-aged populations), an alpha level of 0.05, and statistical power (1-β) of 0.80 were specified. The analysis indicated that a minimum of 72 students would be required. To account for possible attrition due to absence, illness, or withdrawal, the recruitment target was set at a minimum of 90 participants.

Inclusion criteria were regular school attendance, medical clearance for participation in non-competitive physical activity, and signed parental consent with student assent. Exclusion criteria included medical conditions that contraindicated physical activity, severe learning or behavioral disorders requiring individualized curricula incompatible with the standardized testing procedures, and participation in fewer than 80% of intervention sessions during any given phase.

Randomization occurred at the class level to avoid contamination within peer groups. Each class was randomly assigned to one of three possible intervention sequences according to a Latin Square design, balancing order effects: Sequence 1 (morning–midday–afternoon; TA→TB→TC), Sequence 2 (midday–afternoon–morning; TB→TC→TA), and Sequence 3 (afternoon–morning–midday; TC→TA→TB). Allocation was performed by an independent researcher using computer-generated random numbers. Teachers and school administrators were informed of the assigned sequence prior to the start of the intervention.

Final enrollment included 102 students (51 females, 51 males; mean age = 12.6 ± 0.4 years), distributed across eight classes from the two schools. Participants were drawn from two public schools in Southern Italy and reflected the socio-economic diversity of their local school communities. Attendance and adherence to the sessions were systematically monitored, and only students who attended at least 80% of the planned sessions were included in per-protocol analyses, while all enrolled participants were retained in the intention-to-treat analyses.

### 2.3. Procedures

Prior to the start of the study, information sessions were organized with school administrators, teachers, students, and families to explain the objectives, procedures, and requirements of participation. Baseline assessments were conducted during the first week of January 2025, including demographic data collection, health screening, and initial cognitive testing.

Following enrollment, each class was randomly assigned to one of three intervention sequences according to a Latin Square design. The intervention phases consisted of four consecutive weeks of structured physical activity carried out twice per week, always at the same circadian timing assigned to that condition (08:10–09:10; 12:10–13:10; or 15:10–16:10). Each session lasted one school hour and included warm-up, aerobic circuit training at moderate intensity, and cool-down. All sessions were conducted by trained physical education teachers with support from research staff who monitored adherence and recorded attendance. Between each intervention phase, a one-week washout was scheduled in which no experimental sessions were conducted and students continued with their regular physical education curriculum.

Cognitive assessments were administered at two levels. First, acute effects were measured by administering brief tests of attention, working memory, and processing speed immediately before and after selected activity sessions in the second and fourth weeks of each intervention phase. Accordingly, each phase included two acute assessment days (Weeks 2 and 4), with one pre-test and one post-test administered around the same lesson, resulting in a total of six acute assessment days and twelve acute test administrations per participant across the three intervention phases. Second, longitudinal effects were assessed at baseline and at the end of each four-week condition, with repeated administration of the full test battery. These four longitudinal assessments (T0–T3) were distinct from the acute measures and captured cumulative effects. To ensure comparability and replicability of long-term outcomes, all longitudinal assessments (T0–T3) were consistently scheduled in the morning, between 8:30 and 10:00 a.m., across all conditions. By contrast, the acute assessments were strictly tied to the timing of the respective activity sessions. Subjective alertness, sleep quality, and chronotype were also recorded using validated questionnaires.

All data collection was carried out during regular school hours under standardized conditions. Test administrators were trained research assistants, independent from the school staff, whose role was limited to the standardized administration and scoring of the cognitive tasks. Although they were necessarily aware of whether a test was conducted before or after a physical activity session, they were blinded to the sequence of circadian timing conditions (morning, midday, afternoon) to which each class had been allocated and were not informed about the study hypotheses regarding timing effects. Data were anonymized using alphanumeric codes and stored securely in password-protected electronic files. Students who missed more than 20% of the sessions in a given phase were excluded from per-protocol analyses but remained in the intention-to-treat dataset.

### 2.4. Physical Activity Intervention

The physical activity intervention (Table 2) was standardized across all timing conditions to ensure that the only variable under investigation was the circadian placement of the sessions. Each session lasted 60 min and was delivered twice per week over a four-week period, for a total of eight sessions per condition. The sessions were conducted by trained physical education teachers, with research staff monitoring fidelity, attendance, and exercise intensity.

Each class session lasted 60 min, corresponding to a standard school lesson, and was structured into four parts: (1) a ten-minute warm-up consisting of dynamic mobility and light aerobic movements, (2) thirty to thirty-five minutes of circuit-based aerobic training at moderate intensity, (3) ten minutes of cool-down with walking and static stretching, and (4) in selected sessions, brief cognitive testing immediately before and after the activity. The target intensity was set at 60–75% of estimated HRmax, monitored via the OMNI-RPE scale for children (target 5–7 out of 10).

The intervention was designed with progressive difficulty across the four weeks, beginning with familiarization and basic conditioning and culminating in mixed endurance–agility formats. Exercises were varied weekly to sustain motivation, ensure engagement, and stimulate different aspects of motor performance while maintaining comparable aerobic load across conditions.

### 2.5. Measures

A battery of standardized and psychometrically validated instruments was used to assess cognitive performance, subjective alertness, and relevant moderating variables.

#### 2.5.1. Primary Cognitive Outcomes

Selective and sustained attention were measured using the d2-R Test of Attention [26], which provides standardized indices of processing speed, concentration performance, and accuracy. Working memory was assessed through the Digit Span backward subtest from the WISC-IV [27], a widely used measure of executive working memory capacity. Processing speed and visual scanning were evaluated using the Trail Making Test—Part A (TMT-A) [28], which requires participants to connect numbered circles as quickly and accurately as possible.

#### 2.5.2. Secondary Outcomes

Subjective alertness and sleepiness were assessed using the Karolinska Sleepiness Scale (KSS) [29], a validated 9-point Likert scale ranging from “extremely alert” to “very sleepy, struggling to stay awake.” Mood states were evaluated with the short version of the Positive and Negative Affect Schedule for Children (PANAS-C) [30], which provides indices of positive and negative affect and has been validated in adolescent populations.

#### 2.5.3. Moderators and Control Variables

Chronotype was assessed at baseline using the Morningness–Eveningness Questionnaire for Children and Adolescents (MEQ-C) [31], while habitual sleep patterns were measured using a 7-day sleep diary complemented by parental reporting. General physical activity habits outside the school setting were evaluated with the Physical Activity Questionnaire for Adolescents (PAQ-A) [32], a self-report instrument validated for this age group. Anthropometric measures (height, weight, and BMI) were collected at baseline by trained staff following standardized procedures.

#### 2.5.4. Assessment Schedule

Primary and secondary outcomes were assessed both acutely (immediately before and after selected intervention sessions in Weeks 2, 4, 7, 9, 12, and 14) and longitudinally (at baseline T0 and at the end of each four-week intervention phase: T1, T2, T3). Moderators and background measures were collected only at baseline.

### 2.6. Statistical Analysis

Data analysis was conducted using SPSS v.29 (IBM Corp., Armonk, NY, USA) and R v.4.3.3. Descriptive statistics were calculated for all variables and presented as means and standard deviations for continuous data and as frequencies and percentages for categorical data. Normality was checked through the Shapiro–Wilk test and Q–Q plots. Outliers were identified using standardized residuals (>|3.0|) and Winsorized when necessary.

To evaluate longitudinal effects, repeated-measures analyses were carried out using linear mixed-effects models (LMMs), which account for the hierarchical structure of the data (students nested within classes) and for missing values under the assumption of missing at random. Fixed effects included Timing Condition (morning, midday, afternoon), Time (baseline, T1, T2, T3), and their interaction, while Class and Participant were specified as random intercepts. Sex, BMI, chronotype (MEQ-C score), and average sleep duration were included as covariates in the models. Post hoc comparisons were adjusted using the Holm–Bonferroni correction. Effect sizes were expressed as Hedges’ g for pairwise contrasts and partial η^2^ for omnibus tests.

To examine acute effects, pre–post changes in cognitive outcomes (d2-R, Digit Span backward, TMT-A) and subjective alertness (KSS) within each timing condition were analyzed using two-way repeated-measures ANOVAs with the factors Condition (morning, midday, afternoon) and Time (pre vs. post). Where sphericity assumptions were violated, Greenhouse–Geisser corrections were applied. Paired-samples t-tests with Holm–Bonferroni correction were used for planned within-condition contrasts. Because the study employed a three-period crossover design, classes were randomized to one of three intervention sequences according to a Latin Square, which ensured balance of order effects. One-week washout periods between phases were introduced to minimize potential carry-over. Although randomization was implemented at the class level, analyses were performed at the individual level, consistent with previous school-based crossover trials with a limited number of clusters.

Mood states (PANAS-C) were analyzed with mixed ANOVAs including the within-subject factor Timing Condition and the between-subject factor Sex, given the evidence for sex differences in affective responses.

Exploratory analyses were conducted to test for potential moderation effects of chronotype (morning vs. evening types) on the relationship between timing of activity and cognitive outcomes using interaction terms in the LMMs.

The significance threshold was set at *p* < 0.05 for all tests. Confidence intervals (95% CI) were reported where applicable.

## 3. Results

### 3.1. Longitudinal Effects of Physical Activity Timing: Linear Mixed-Effects Models (LMMs)

#### 3.1.1. Primary Cognitive Outcomes

**Attention (d2-R)**

Linear mixed-effects models revealed a significant main effect of Timing Condition (F(2, 196) = 18.7, *p* < 0.001, η^2^p = 0.16) and of Time (F(3, 294) = 12.4, *p* < 0.001, η^2^p = 0.11). Interaction effects were also significant (F(6, 588) = 4.9, *p* < 0.001, η^2^p = 0.05). Post hoc comparisons showed that morning sessions (298.3 ± 21.1) produced higher concentration scores compared with midday (293.1 ± 20.9; *p* = 0.012, g = 0.25) and afternoon sessions (288.3 ± 21.8; *p* < 0.001, g = 0.45). Scores improved across time points, from baseline (280.4 ± 19.7) to T3 (295.6 ± 20.8).

**Working memory (Digit Span backward)**

A significant main effect of Timing Condition was found (F(2, 196) = 6.8, *p* = 0.002, η^2^p = 0.06), with morning sessions (7.9 ± 1.6) outperforming afternoon sessions (7.3 ± 1.4; *p* = 0.001, g = 0.38). The main effect of Time was also significant (F(3, 294) = 9.2, *p* < 0.001, η^2^p = 0.08), showing gradual improvements from baseline (7.2 ± 1.5) to T3. The interaction effect was significant (F(6, 588) = 3.1, *p* = 0.005, η^2^p = 0.03), with the largest gains observed in the morning condition at later time points.

**Processing speed (TMT-A)**

Completion times were significantly influenced by Timing Condition (F(2, 196) = 9.5, *p* < 0.001, η^2^p = 0.08) and Time (F(3, 294) = 14.6, *p* < 0.001, η^2^p = 0.13). The Timing × Time interaction was also significant (F(6, 588) = 2.8, *p* = 0.012, η^2^p = 0.03). Morning sessions (29.1 ± 5.7 s) produced faster completion compared to midday (30.5 ± 5.9 s; *p* = 0.041, g = 0.22) and afternoon sessions (31.2 ± 6.2 s; *p* < 0.001, g = 0.35).

#### 3.1.2. Secondary Outcomes

**Subjective alertness (KSS)**

KSS scores differed significantly by Timing Condition (F(2, 196) = 22.9, *p* < 0.001, η^2^p = 0.19), with lower sleepiness in the morning (3.4 ± 1.1) compared to midday (4.1 ± 1.2) and afternoon (4.6 ± 1.3). No significant effects of Time (*p* = 0.21) or interaction (*p* = 0.12) were found.

**Mood (PANAS-C)**

For positive affect, there was a significant main effect of Timing Condition (F(2, 196) = 6.1, *p* = 0.003, η^2^p = 0.05), with higher scores following morning (28.6 ± 6.3) and midday sessions (27.4 ± 6.1) compared with afternoon (25.7 ± 6.4). A significant effect of Time was also found (F(3, 294) = 4.7, *p* = 0.003, η^2^p = 0.05). The interaction effect was not significant (*p* = 0.09). For negative affect, no significant main effects or interactions were detected (all *p* > 0.30).

The following Table 3 illustrates the results for primary and secondary outcomes according to Linear Mixed-Effects Models:

### 3.2. Acute Pre–Post Effects of School-Based Physical Activity: Two-Way Repeated-Measures ANOVAs

**Attention (d2-R)**

Two-way repeated-measures ANOVA revealed a significant main effect of Time (F(1, 101) = 42.5, *p* < 0.001, η^2^p = 0.30) and Condition (F(2, 202) = 11.8, *p* < 0.001, η^2^p = 0.10), as well as a significant Condition × Time interaction (F(2, 202) = 6.4, *p* = 0.002, η^2^p = 0.06). Post hoc paired-samples t-tests showed that d2-R scores improved significantly pre–post in all conditions, with the largest effect in the morning (Δ = +15.2 points, *p* < 0.001, g = 0.62), followed by midday (Δ = +11.1, *p* < 0.001, g = 0.45), and afternoon (Δ = +7.8, *p* = 0.004, g = 0.32).

**Working memory (Digit Span backward)**

A main effect of Time was observed (F(1, 101) = 18.9, *p* < 0.001, η^2^p = 0.16), with post-intervention scores higher across conditions. The Condition × Time interaction was significant (F(2, 202) = 3.7, *p* = 0.026, η^2^p = 0.04). Morning activity yielded the largest acute improvement (Δ = +0.6, *p* < 0.001, g = 0.40), midday showed moderate gains (Δ = +0.4, *p* = 0.012, g = 0.25), while afternoon changes were not significant after correction (Δ = +0.2, *p* = 0.091).

**Processing speed (TMT-A)**

A main effect of Time was found (F(1, 101) = 24.6, *p* < 0.001, η^2^p = 0.20), with faster completion times post-intervention. The Condition × Time interaction was significant (F(2, 202) = 4.2, *p* = 0.016, η^2^p = 0.04). Paired-samples comparisons showed significant pre–post improvement in morning (Δ = –2.9 s, *p* < 0.001, g = 0.35) and midday conditions (Δ = –2.1 s, *p* = 0.004, g = 0.28), but not in the afternoon (Δ = –1.0 s, *p* = 0.087).

**Subjective alertness (KSS)**

Significant effects of Time (F(1, 101) = 29.7, *p* < 0.001, η^2^p = 0.23), Condition (F(2, 202) = 13.5, *p* < 0.001, η^2^p = 0.12), and their interaction (F(2, 202) = 5.8, *p* = 0.004, η^2^p = 0.05) were detected. Morning activity produced the largest reduction in sleepiness (Δ = –0.8 points, *p* < 0.001, g = 0.48), followed by midday (Δ = –0.5, *p* = 0.018, g = 0.30). Afternoon reductions were smaller and non-significant after correction (Δ = –0.3, *p* = 0.079).

The following Table 4 illustrates the results concerning the acute pre–post effects of school-based physical activity according to two-way repeated-measures ANOVAs.

### 3.3. Sex Differences in Affective Responses: Mixed ANOVAs

**Positive affect (PANAS-C)**

Mixed ANOVA showed a significant main effect of Timing Condition (F(2, 200) = 6.4, *p* = 0.002, η^2^p = 0.06), with higher positive affect after morning (28.6 ± 6.3) and midday activity (27.4 ± 6.1) compared to afternoon sessions (25.7 ± 6.4). A main effect of Sex was also significant (F(1, 100) = 5.8, *p* = 0.018, η^2^p = 0.05), with females reporting overall lower positive affect (M = 26.4 ± 6.2) compared to males (M = 28.3 ± 6.4). The Timing × Sex interaction was not significant (F(2, 200) = 1.2, *p* = 0.29).

**Negative affect (PANAS-C)**

For negative affect, no significant main effects of Timing Condition (F(2, 200) = 0.9, *p* = 0.41) or Sex (F(1, 100) = 2.1, *p* = 0.15) were observed, nor a significant interaction (F(2, 200) = 0.6, *p* = 0.55). Scores remained stable across sessions and between groups, averaging 13.5 ± 4.2 overall.

The following Table 5 illustrates the results concerning sex differences in affective responses according to mixed ANOVAs:

### 3.4. Chronotype as a Moderator of Timing Effects: Exploratory LMM Analyses

Exploratory linear mixed-effects models including the Timing × Chronotype interaction showed partial evidence that chronotype moderated the relationship between activity timing and cognitive performance.

**Attention (d2-R)**

A significant Timing × Chronotype interaction was observed (F(2, 194) = 4.6, *p* = 0.012, η^2^p = 0.05). Morning-type students showed greater benefits from morning sessions (Δ = +20.5 points compared to baseline) than from afternoon sessions (Δ = +8.3), whereas evening-type students exhibited smaller differences (morning Δ = +12.1, afternoon Δ = +9.6).

**Working memory (Digit Span backward)**

The interaction effect was also significant (F(2, 194) = 3.9, *p* = 0.022, η^2^p = 0.04). Morning-type students improved more after morning activity (Δ = +0.8) than after afternoon activity (Δ = +0.2, ns), while evening types showed similar improvements across timings (Δ = +0.4–0.5).

**Processing speed (TMT-A)**

The Timing × Chronotype interaction did not reach significance (F(2, 194) = 2.1, *p* = 0.11). Both chronotype groups improved after physical activity, with morning types showing slightly greater improvements in the morning condition (Δ = –3.2 s) compared to evening types (Δ = –2.1 s), but differences were not statistically significant.

Overall, the findings suggest that chronotype moderated the acute and longitudinal benefits of morning activity for attention and working memory, whereas effects on processing speed were more consistent across chronotype groups.

The following Table 6 illustrates the results concerning chronotype as a moderator of timing effects according to exploratory LMM analyses.

## 4. Discussion

The present study investigated the role of circadian timing in shaping the cognitive and affective benefits of school-based physical activity among early adolescents. By systematically comparing morning, midday, and afternoon sessions in a crossover design, our findings provide novel evidence that the timing of activity within the school day significantly modulates its impact on attention, working memory, processing speed, subjective alertness, and mood states. The results are consistent with previous studies indicating that morning activity produces the most robust improvements, midday sessions show moderate effects, and afternoon sessions yield the weakest or nonsignificant changes [33,34,35]. These outcomes carry important implications for both educational practice and the broader field of chronobiology in exercise science.

A central finding of the study is that attentional performance, as measured by the d2-R test, improved most strongly after morning physical activity. This is consistent with previous literature suggesting that early-day activity aligns with optimal phases of the circadian rhythm in adolescents, when cortisol levels and core body temperature are rising and cognitive readiness is enhanced [36,37]. The significant Timing × Time interaction further indicates that the benefits of morning exercise were not only acute but also accumulated across the intervention phases. Midday activity also produced meaningful but smaller improvements, while afternoon activity was associated with the lowest performance levels. Taken together, these results suggest that the alignment of exercise with the early morning hours maximizes attentional benefits, likely by enhancing arousal regulation and executive control. Previous studies have suggested that hormonal and thermoregulatory changes, including fluctuations in cortisol and core body temperature, may contribute to these effects, although such mechanisms were not directly measured in the present study [38].

Working memory, assessed by the Digit Span backward task, followed a similar pattern. Morning sessions produced the greatest gains, with significant pre–post increases and longitudinal improvements across the intervention. Midday sessions yielded moderate but consistent effects, whereas afternoon sessions produced limited changes. This pattern may reflect the interplay between circadian fluctuations in prefrontal cortical functioning and the cognitive demands of working memory tasks. Adolescents are known to exhibit delayed circadian rhythms relative to adults, but our results suggest that the early morning session (8:10–9:10) represents a particularly favorable window for enhancing working memory performance through exercise [39]. Afternoon periods, by contrast, may coincide with circadian dips in alertness and reduced executive functioning, attenuating the benefits of activity [40].

Processing speed, measured by the Trail Making Test, also demonstrated stronger improvements in the morning condition. Students completed the task significantly faster after morning activity compared to midday and afternoon sessions, with interaction effects showing that these benefits accumulated over time. Given that processing speed relies on both psychomotor activation and efficient visual scanning, the superior morning outcomes may be attributable to higher physiological arousal and reduced mental fatigue during the early school hours. These results resonate with prior work linking exercise-induced increases in catecholamines with improved psychomotor functioning, particularly when performed at times aligned with endogenous circadian peaks [41,42].

Subjective alertness, measured by the Karolinska Sleepiness Scale, revealed a clear circadian gradient. Morning activity produced the largest reductions in sleepiness, midday activity led to moderate decreases, and afternoon activity was least effective. Interestingly, the effect of time across intervention weeks was not significant, suggesting that these patterns are stable and not subject to habituation. The implication is that physical activity operates as an acute modulator of alertness, with greater efficacy when scheduled early in the day. This finding is particularly relevant for educational practice, as it demonstrates that exercise can counteract morning lethargy in adolescents and prepare them for cognitively demanding tasks [43].

Mood states, assessed through the PANAS-C, showed partially convergent patterns. Positive affect was significantly higher after morning and midday sessions compared to afternoon sessions, while negative affect remained stable across conditions. The sex effect observed for positive affect, with males reporting slightly higher levels than females, is consistent with prior literature indicating sex-based differences in affective responses to exercise. Importantly, the absence of significant interactions suggests that the benefits of morning activity on positive affect are broadly shared across sexes. These results reinforce the view that physical activity scheduled earlier in the day contributes to both cognitive readiness and emotional well-being in adolescents [44,45].

Exploratory moderation analyses provided further nuance by demonstrating that chronotype influenced the magnitude of timing effects. Morning-type students derived the largest benefits from morning sessions in both attention and working memory, while evening-type students showed attenuated differences across conditions. This aligns with circadian theory, which posits that individual chronotype modulates the alignment between environmental demands and endogenous biological rhythms [46]. The absence of significant interactions for processing speed suggests that some domains may be less sensitive to chronotype, or that larger samples are needed to detect such effects [47]. Nonetheless, these exploratory findings highlight the importance of personalizing interventions based on circadian profiles, an approach that could enhance inclusivity and effectiveness in school contexts.

From a pedagogical perspective, the findings have practical implications for curriculum planning and school health promotion. The consistent superiority of morning sessions suggests that integrating structured physical activity into the early part of the school day could serve as a catalyst for improved academic readiness [48]. Such an approach aligns with recent calls to reorganize school timetables in ways that are biologically informed, respecting circadian principles while also addressing the developmental needs of adolescents. Importantly, the observed benefits were not limited to cognitive outcomes but extended to subjective alertness and positive affect, dimensions that play a crucial role in student engagement and classroom climate.

Our findings also contribute to the growing literature on the intersection of chronobiology and educational neuroscience. While numerous studies have demonstrated the cognitive benefits of physical activity, relatively few have examined the moderating role of circadian timing in real-world school settings [49,50,51,52,53,54,55]. By adopting a crossover design and including both acute and longitudinal measures, the present study advances this line of inquiry and provides a more granular understanding of how exercise interacts with temporal dynamics to shape cognitive and affective outcomes.

Nevertheless, several limitations must be acknowledged. First, although the crossover design strengthens internal validity, residual carryover effects between conditions cannot be completely ruled out, even with washout periods. Second, the sample was drawn from a specific geographical context in southern Italy, which may limit the generalizability of findings to other cultural or educational systems. Third, while standardized cognitive tests were employed, the classroom setting introduces ecological variability that may have influenced performance. Finally, chronotype analyses were exploratory, and future studies with larger samples and objective measures of circadian phase are needed to validate these findings.

Future research should expand on the present results by exploring interactions between exercise intensity, circadian timing, and cognitive domains. It would also be valuable to integrate objective measures of physiological arousal, such as heart rate variability or cortisol levels, to better elucidate the mechanisms linking exercise timing and cognitive performance. Moreover, longitudinal studies spanning entire school years could determine whether scheduling physical activity in the morning yields sustained academic benefits and whether such effects translate into measurable improvements in academic achievement.

Overall, this study demonstrates that the timing of school-based physical activity is a critical determinant of its cognitive and affective outcomes in adolescents. Morning sessions consistently produced the strongest benefits across multiple domains, including attention, working memory, processing speed, alertness, and positive affect. These results underscore the value of adopting circadian-informed approaches in educational planning and highlight the potential of physical activity as a strategic tool for optimizing both learning and well-being in schools.

## 5. Conclusions

The present study addressed the question of whether the timing of school-based physical activity influences its cognitive and emotional effects in adolescents. By comparing morning, midday, and afternoon sessions in a controlled crossover design, the findings provide evidence that circadian alignment contributes significantly to both acute and longitudinal outcomes.

Overall, morning sessions were associated with the most consistent improvements in attention, working memory, processing speed, subjective alertness, and positive affect, whereas midday sessions produced moderate benefits and afternoon sessions the weakest or nonsignificant effects. These results indicate that the effectiveness of school-based physical activity is not uniform but depends on when it is scheduled within the school day.

From a practical standpoint, the evidence suggests that prioritizing morning sessions may offer a feasible and low-cost approach to enhance learning readiness and emotional well-being. However, given the variability introduced by factors such as chronotype and sleep patterns, these findings should be applied cautiously in policy and curricular decisions. Schools considering timetable adjustments may benefit from pilot implementations before broader adoption.

Future research should build on these results by testing different combinations of exercise intensity, duration, and timing, and by incorporating objective physiological markers to clarify underlying mechanisms. Longer-term studies are needed to evaluate whether the observed benefits persist across entire school years and translate into academic achievement. Moreover, examining interindividual variability will be essential to developing flexible, evidence-based recommendations that maximize both effectiveness and inclusivity.

## Figures and Tables

**Table 1:** Characteristics of the Sample (N = 102).

Variable	Total (N = 102)	Males (n = 51)	Females (n = 51)
Age (years), mean ± SD	12.6 ± 0.4	12.6 ± 0.4	12.7 ± 0.3
Age range (years)	12–13	12–13	12–13
Body weight (kg), mean ± SD	47.8 ± 8.5	48.9 ± 8.7	46.7 ± 8.2
Height (cm), mean ± SD	156.2 ± 7.1	157.3 ± 6.9	155.1 ± 7.3
BMI (kg/m^2^), mean ± SD	19.6 ± 2.7	19.8 ± 2.8	19.4 ± 2.6
Resting heart rate (bpm), mean ± SD	78.5 ± 6.9	77.9 ± 7.1	79.1 ± 6.7
Extended school schedule (%)	100	100	100
Students meeting ≥ 80% attendance (%)	94 (92%)	47 (92%)	47 (92%)

*Note: Values are expressed as mean ± standard deviation unless otherwise indicated.*

**Table 2:** Weekly Intervention Protocol.

Week	Structure & Progression	Exercises (Examples)	Work-to-Rest Ratio
1—Familiarization	Introduction to circuit training, focus on technique and pacing	Shuttle runs (10–15 m), jumping jacks, bodyweight squats, plank hold, high-knee runs on the spot, cone weaving	40 s work/30 s transition × 2 rounds
2—Progressive Load	Increased duration, introduction of directional changes and dynamic movements	Shuttle runs with changes in direction, star jumps, dynamic squats with arm swings, plank with alternating knee-to-elbow, high-knee runs, zig-zag sprints between cones	45 s work/30 s transition × 2 rounds
3—Consolidation	Reduced transition times, focus on coordination and rhythm	Skipping with rope, squat jumps, side shuffles, plank with shoulder taps, walking lunges, agility ladder drills (or equivalent markers)	45 s work/25 s transition × 2 rounds
4—Mixed Circuit & Endurance	Combination of circuit and continuous run for adaptation	Round 1: shuttle runs, mountain climbers, squat jumps, plank-to-push-up, lateral cone hops, fast-feet drill; Continuous run: 6 min shuttle run at moderate pace; Round 2: same circuit repeated	Circuit: 45 s work/25 s transition; Continuous run: 6 min

**Table 3:** Mean scores of cognitive tests across circadian timing conditions. Baseline (T0) and longitudinal assessments (T1–T3) were administered in the morning between 08:30 and 10:00 a.m. to ensure comparability across conditions. Morning, midday, and afternoon values refer to acute cognitive tests conducted immediately before and after the scheduled activity sessions at the corresponding circadian timing (morning: 08:10–09:10; midday: 12:10–13:10; afternoon: 15:10–16:10). Within each intervention phase, acute assessments were performed in Weeks 2 and 4, and the two measurements were averaged to obtain a single value per timing condition for each participant.

Outcome	Baseline	Morning	Midday	Afternoon	F (df)	*p*	η^2^p
Primary cognitive outcomes							
d2-R (concentration)	280.4 ± 19.7	298.3 ± 21.1	293.1 ± 20.9	288.3 ± 21.8	18.7 (2, 196)	<0.001	0.16
Digit Span backward	7.2 ± 1.5	7.9 ± 1.6	7.6 ± 1.5	7.3 ± 1.4	6.8 (2, 196)	0.002	0.06
TMT-A (s) ↓ better	32.8 ± 6.3	29.1 ± 5.7	30.5 ± 5.9	31.2 ± 6.2	9.5 (2, 196)	<0.001	0.08
Secondary outcomes							
KSS (lower = more alert)	—	3.4 ± 1.1	4.1 ± 1.2	4.6 ± 1.3	22.9 (2, 196)	<0.001	0.19
PANAS-C Positive Affect	—	28.6 ± 6.3	27.4 ± 6.1	25.7 ± 6.4	6.1 (2, 196)	0.003	0.05
PANAS-C Negative Affect	—	13.1 ± 4.2	13.5 ± 4.1	13.8 ± 4.3	0.9 (2, 196)	0.42	0.01

*Note*. *Values are mean ± SD. F, p, and η^2^p values refer to the main effect of Timing Condition from linear mixed-effects models adjusted for sex, BMI, chronotype, and sleep duration. Baseline values are provided only for cognitive outcomes (d2-R, Digit Span backward, TMT-A); secondary outcomes (KSS, PANAS-C) were measured only at intervention sessions.* ↓ *indicates that lower values reflect better performance.*

**Table 4:** Acute effects (pre–post) across timing conditions.

Outcome	Morning Pre	Morning Post	Midday Pre	Midday Post	Afternoon Pre	Afternoon Post	Interaction *p*
d2-R (concentration)	283.1 ± 20.4	298.3 ± 21.1	282.0 ± 19.9	293.1 ± 20.9	280.5 ± 20.1	288.3 ± 21.8	0.002
Digit Span backward	7.3 ± 1.4	7.9 ± 1.6	7.2 ± 1.5	7.6 ± 1.5	7.1 ± 1.4	7.3 ± 1.4	0.026
TMT-A (s) ↓ better	32.0 ± 6.1	29.1 ± 5.7	32.6 ± 6.3	30.5 ± 5.9	33.0 ± 6.5	31.9 ± 6.2	0.016
KSS (lower = more alert)	4.2 ± 1.2	3.4 ± 1.1	4.6 ± 1.3	4.1 ± 1.2	4.9 ± 1.4	4.6 ± 1.3	0.004

*Note. Values are mean ± SD. Interaction p-values refer to the Condition × Time effect in two-way repeated-measures ANOVA. Pre–post contrasts were adjusted with Holm–Bonferroni correction.*↓ *indicates that lower values reflect better performance*.

**Table 5:** Mood states (PANAS-C) across timing conditions and sex.

Outcome	Morning	Midday	Afternoon	Main Effect (Timing)	Main Effect (Sex)	Interaction
Positive Affect (Males)	29.5 ± 6.1	28.3 ± 6.0	26.9 ± 6.2	F(2, 200) = 6.4, *p* = 0.002, η^2^p = 0.06	F(1, 100) = 5.8, *p* = 0.018, η^2^p = 0.05	ns
Positive Affect (Females)	27.7 ± 6.4	26.5 ± 6.2	24.5 ± 6.5			
Negative Affect (Males)	13.0 ± 4.1	13.3 ± 4.0	13.5 ± 4.3	F(2, 200) = 0.9, *p* = 0.41	F(1, 100) = 2.1, *p* = 0.15	ns
Negative Affect (Females)	13.2 ± 4.3	13.6 ± 4.2	14.0 ± 4.4			

*Note. Values are mean ± SD. ns = non significant. Positive affect scores were higher in morning and midday sessions compared to afternoon, with males consistently reporting higher positive affect than females. Negative affect did not vary significantly across timing or sex.*

**Table 6:** Chronotype as moderator of timing effects on cognitive outcomes.

Outcome	Morning Types (n = 23)	Evening Types (n = 42)	F (df)	*p*	η^2^p
d2-R (concentration) Δ	+20.5 ± 9.4	+12.1 ± 8.7	4.6 (2, 194)	0.012	0.05
Digit Span backward Δ	+0.8 ± 0.5	+0.4 ± 0.4	3.9 (2, 194)	0.022	0.04
TMT-A (s) Δ ↓ better	–3.2 ± 2.5	–2.1 ± 2.3	2.1 (2, 194)	0.11	0.02

*Note. Values represent mean change scores (Δ) from baseline. Interaction statistics refer to the Timing × Chronotype effect in LMMs, adjusted for sex, BMI, and sleep duration. Significant moderation was found for d2-R and Digit Span backward, but not for TMT-A.* ↓ *indicates that lower values reflect better performance.*

## Data Availability

The data presented in this study are available on request from the corresponding author. The data are not publicly available due to privacy restrictions.
